# MelLec Exacerbates the Pathogenesis of *Aspergillus fumigatus*-Induced Allergic Inflammation in Mice

**DOI:** 10.3389/fimmu.2021.675702

**Published:** 2021-05-28

**Authors:** Kazuya Tone, Mark H. T. Stappers, Remi Hatinguais, Ivy M. Dambuza, Fabián Salazar, Carol Wallace, Raif Yuecel, Petruta L. Morvay, Kazuyoshi Kuwano, Janet A. Willment, Gordon D. Brown

**Affiliations:** ^1^ Aberdeen Fungal Group, Institute of Medical Sciences, University of Aberdeen, Aberdeen, United Kingdom; ^2^ Division of Respiratory Diseases, Department of Internal Medicine, The Jikei University School of Medicine, Tokyo, Japan; ^3^ Medical Research Council Centre for Medical Mycology at the University of Exeter, Exeter, United Kingdom; ^4^ Iain Fraser Cytometry Centre, Institute of Medical Sciences, University of Aberdeen, Aberdeen, United Kingdom; ^5^ Exeter Centre for Cytomics (EXCC), Department of Biosciences, College of Life and Environmental Sciences, University of Exeter, Exeter, United Kingdom

**Keywords:** C-type lectin, asthma, *Aspergillus fumigatus*, MelLec, allergy

## Abstract

Environmental factors, particularly fungi, influence the pathogenesis of allergic airway inflammation, but the mechanisms underlying these effects are still unclear. Melanin is one fungal component which is thought to modulate pulmonary inflammation. We recently identified a novel C-type lectin receptor, MelLec (Clec1a), which recognizes fungal 1,8-dihydroxynaphthalene (DHN)-melanin and is able to regulate inflammatory responses. Here we show that MelLec promotes pulmonary allergic inflammation and drives the development of Th17 T-cells in response to spores of *Aspergillus fumigatus*. Unexpectedly, we found that MelLec deficiency was protective, with MelLec^-/-^ animals showing normal weight gain and significantly reduced pulmonary inflammation in our allergic model. The lungs of treated MelLec^-/-^ mice displayed significantly reduced inflammatory foci and reduced bronchial wall thickening, which correlated with a reduced cellular influx (particularly neutrophils and inflammatory monocytes) and levels of inflammatory cytokines and chemokines. Notably, fungal burdens were increased in MelLec^-/-^ animals, without apparent adverse effects, and there were no alterations in the survival of these mice. Characterization of the pulmonary T-cell populations, revealed a significant reduction in Th17 cells, and no alterations in Th2, Th1 or Treg cells. Thus, our data reveal that while MelLec is required to control pulmonary fungal burden, the inflammatory responses mediated by this receptor negatively impact the animal welfare in this allergic model.

## Introduction

Fungi have an enormous, but largely unrecognized, impact on human morbidity and mortality including allergic airway disease, for which fungal sensitization is increasingly being realized to play a major role in disease pathology (1). There is compelling evidence that fungi are associated with the development and severity of asthma, in particular, and several studies have demonstrated the beneficial effects of antifungal therapy on disease progression (2). Common fungal species associated with sensitization include spp. of *Alternaria*, *Cladosporium* and *Aspergillus* which all possess allergens, including fungal cell wall components, that are recognized by pattern recognition receptors such as those of the C-type lectin receptor (CLR) family (3). CLRs and their downstream signaling pathways play critical roles in mediating innate and adaptive anti-fungal immune responses (4). Moreover, several of these receptors have been implicated in asthma pathogenesis (3, 5).

We recently identified a novel fungal-sensing CLR, MelLec, which recognizes DHN-melanin, a non-carbohydrate cell wall component found in many asthma-associated fungal species, including species of *Aspergillus fumigatus* (6). MelLec is ubiquitously expressed by CD31^+^ endothelial cells in mice, and is also expressed by a small sub-population of these cells that co-express epithelial cell adhesion molecule (EpCAM) that are detected only in the lung and the liver (6). In humans, MelLec is also expressed on myeloid cells (7). We found MelLec to play an essential role during invasive infection with this pathogen in both mice and humans (6), that the receptor is involved in the regulation of early pulmonary inflammatory responses following intratracheal challenge of immunocompetent MelLec-deficient mice with a single bolus of *A. fumigatus* conidia (6). Given the impact of MelLec on pulmonary responses, we explored here the contribution of this CLR to the development of allergic airway inflammation in response to *A. fumigatus* using a murine model.

## Materials and Methods

### Mice

8–10 week old wild type (wt) C57BL/6 and MelLec^-/-^ mice (6) were bred and maintained in the specific pathogen-free facilities of the University of Aberdeen. wt and MelLec^-/-^ animals were co-housed for 2-weeks prior to experimentation and randomly assigned to experimental and control groups. Experiments were not blinded. All animal use was approved and in compliance with local University animal research ethical regulations and a UK Home Office project licence (P79B6F297). Mice were sacrificed by Pentobarbital sodium (Euthatal^®^). The physical condition of the animals was checked at least once daily.

### Airway Challenges

**Figure 1:**
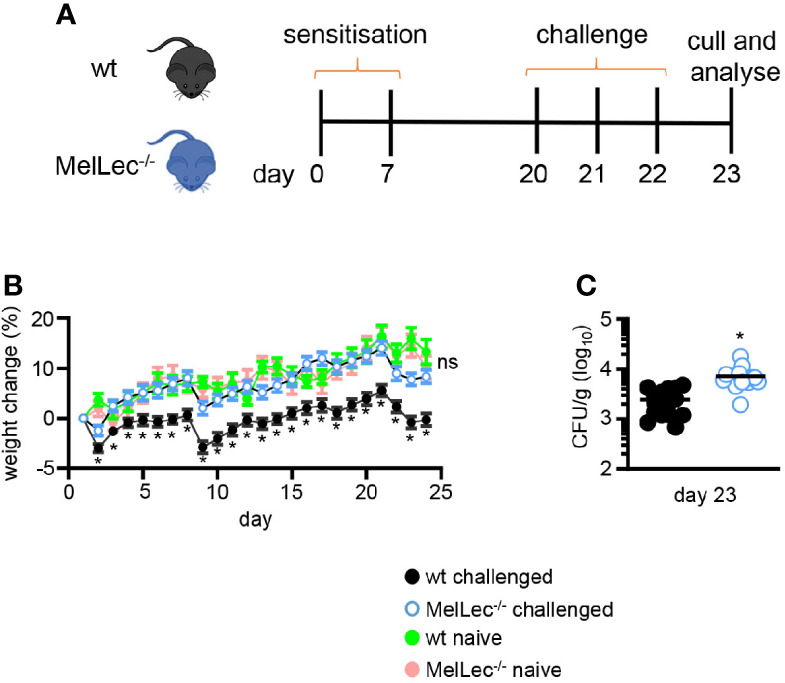
Loss of MelLec protects against body weight loss. **(A)** Schematic representation of the allergic model used in these experiments. Wild type (wt) or MelLec-/- mice were sensitized by intratracheal administration with 1x10^7^ live spores of *Aspergillus fumigatus* ATCC 13073 on days 0 and 7, and then similarly challenged on days 20, 21, and 22. Mice were sacrificed on day 23, 24 hrs after the last-challenge and analyzed. **(B)** Percentage body weight change over the course of the experimental model and naïve Wild type (wt) or MelLec^-/-^ mice, as indicated. Pooled values shown are mean ± SEM of five independent experiments (n>40 mice per group). **(C)** Lung fungal burdens of mice on day 23. CFU, colony-forming unit. Shown are pooled data (with mean) from two independent experiments (n=16 mice). *p<0.05. Statistical significance was determined using t-tests. n.s., not significant.

*Aspergillus fumigatus* ATCC 13073 was cultured on potato dextrose agar plates at 37°C for 7 days and the conidia resuspended in 0.1% of Tween 80, filtered with a 40μm cell strainer to remove mycelia and washed twice in PBS. In all experiments, mice were anesthetized with an intraperitoneal administration of 76 μg/g body weight of ketamine (Narketan^®^) and 1 μg/g body weight of medetomidine (Domitor^®^). Mice were sensitized intratracheally (non-surgical) on days 0, 7 and challenged on days 20, 21, and 22 with *Aspergillus fumigatus* ATCC 13073 conidia at 1x10^7^ in 40 μl of PBS, as per previously established models (5). Mice were roused with an intraperitoneal administration of 1μg/g body weight of atipamezole (Antisedan^®^). Mice were sacrificed on day 23, 24 hrs after the last-challenge with *A. fumigatus* (**Figure 1**). The right lung was used for flow cytometry and the left lung was used for cytokine assays or fungal burdens. *A. fumigatus* conidia were heat-killed at 65°C for 1hr for the data presented in **Supplementary Figure 3**.

### Flow Cytometry

Flow cytometry was performed using standard methodology. In brief, lungs were digested with the lung dissociation kit (Miltenyi Biotec GmbH, Germany) and dispersed using GentleMACS (Miltenyi Biotec GmbH, Germany), and then passed through a 70 μm strainer to obtain single cell suspensions. Cells were recovered by centrifugation (300*g* for 10 mins), red blood cells were lysed using PharmLyse™ (BD Biosciences, USA) and the cells stained with a viability dye (fixable Viability Dye-eFluor780, fixable Viability Dye eFluor 455UV, Fixable Viability Dye eFluor 450; depending on the experiment; all from Thermo Fisher Scientific, USA). Cells were recovered by centrifugation and resuspended in flow staining buffer (0.5% of bovine serum albumin, 2mM of EDTA in sterile PBS) with 8μg/mL of anti-CD16/CD32 (Fc-block, Clone 2.4G2, prepared in house). For analyzing cellular cytokine expression, lung single cell suspensions were plated in 6 well plates at 1x10^6^ cells/well and stimulated with phorbol 12-myristate 13-acetate (PMA) (50 ng/ml), ionomycin (1μg/ml) (both Sigma) at 37°C for 5 hrs. Brefeldin A (5 μg/ml) and Monensin (5 μg/ml) (both Merck KGaA, Germany) were then added, and the cells were incubated for a further 3 hr. For cell surface staining, antibody cocktails containing 1:200 antibodies in PBS were added and incubated for 30 mins on ice. The cells were then washed with staining buffer and analyzed. When required, cells were fixed with 1% paraformaldehyde. For intracellular staining, cells were permeabilized using the Foxp3 permeabilization kit (Thermo Fisher Scientific, USA). The following antibodies were used: CD11B-BUV395 (M1/70) CD11C-BB700 (HL3), CD44-AF700 (IM7), CD44-BB515 (IM7), CD45-BV650 (30-F11), Foxp3-AF647 (MF23), GATA-3-BUV395 (L50-823), IFNγ-FITC (XMG1.2), IL-10-PE (JES5-16E3), IL-17-BV605 (TC11-18H10), IL-4-APC (11B11), Ly6C-FITC (AL-21), Ly6G-PE (1A8), RORγT-PE (Q31-378), T-bet-BUV421 (O4-46) (all from BD) and B220-BV605 (RA3-6B2), CD4-AF700 (RM4-5) (BioLegend, USA). Control samples such as unstained, isotype and Fluorescence Minus One (FMO) were acquired to find out the boundary and specificity of the fluorescence signal. Flow cytometry was performed on BD LSR Fortessa™ (BD Biosciences, USA) or Attune NxT Flow Cytometer (Thermo Fisher Scientific, USA) and analyzed with FlowJo 10 software (BD Biosciences, USA). Gating strategies used are shown in **Supplementary Figures 1** and **2** (8, 9).

### Cytokine Assays and Fungal Burdens

Lungs homogenates were used to assay lung cytokines and fungal burdens. Cytokine levels were analyzed with multiplex bead-based assays (R&D), according to the manufacturer’s instructions. Cytokine concentrations were normalized for protein content (BCA protein assay kit, Thermo Fisher Scientific). For fungal burdens, serially diluted suspensions of aseptically prepared lung homogenates were freshly plated onto potato dextrose agar plates and Colony Forming Units (CFUs) determined after 36-48hrs incubation at 37°C.

### Histology

Lungs were inflated with 50% Optimal Cutting Temperature (OCT, Tissue-Tek, Sakura, Netherlands) compound in PBS followed by fixation in 10% formaldehyde and embedded in paraffin wax. 10μm sections were stained with Haematoxylin and Eosin (H&E) or Periodic Acid Schiff (PAS, TAAB Laboratories, UK) using standard methodology. Quantification of inflammation area, bronchial wall thickening (taking the average of four cross section measurements of each bronchiole), and percentage of PAS positive cells were performed using ZEN2 software (Carl Zeiss) and Image J software (Centre for Information Technology, National Institute of Health, Bethesda, Maryland), scoring five representative bronchioles from at least two different mice. Comparable bronchioles from wt and MelLec^-/-^ mice were selected for analysis, selecting proximal areas that were not adjacent to other bronchioles. Inflammation area and bronchial wall thickening measurements were standardized to the outside diameter of the bronchioles.

### Statistical Analysis

Two-tailed student’s t-tests, Mann–Whitney U tests, or one-way ANOVA were used to determine statistical significance. All experiments were independently repeated at least once, unless otherwise indicated. A *p* value of < 0.05 was considered to be statistically significant. Statistical analyses were performed using Prism (version 7; GraphPad Software, La Jolla, CA, USA).

## Results

### Loss of MelLec Protects Against Body Weight Loss

To determine if MelLec plays a role in the development of pulmonary allergic responses, we made use of a standard mouse model of sensitization and challenge (5, 10) using live *A. fumigatus* conidia (**Figure 1A**). Treatment of wt mice significantly affected their ability to gain weight, compared to untreated animals (**Figure 1B**). In contrast, MelLec-deficient mice displayed no adverse effects, gaining weight at a rate comparable to naïve animals (**Figure 1B**). Moreover, the knockout animals did not display any clinical symptoms or succumb to the repeated conidial challenge (data not shown). The effect on weight gain was not due to any intrinsic difference in the two groups of animals, as weight gain in naïve mice was equivalent (**Figure 1B**), as we reported previously (6). The effect of this protocol in wt animals required live conidia, as administration of heat-killed conidia had no effect on weight gain or inflammation (**Supplementary Figures 3A, B**). Remarkably, despite showing no adverse clinical symptoms, MelLec^-/-^ mice presented with substantially higher pulmonary fungal burdens (**Figure 1C**). Fungal burdens were cleared in both groups of animals 7 days following last challenge. No CFU were detected in the lung of unchallenged animals (not shown).

### MelLec^-/-^ Mice Have Reduced Pulmonary Inflammation and Bronchial Wall Thickening

**Figure 2:**
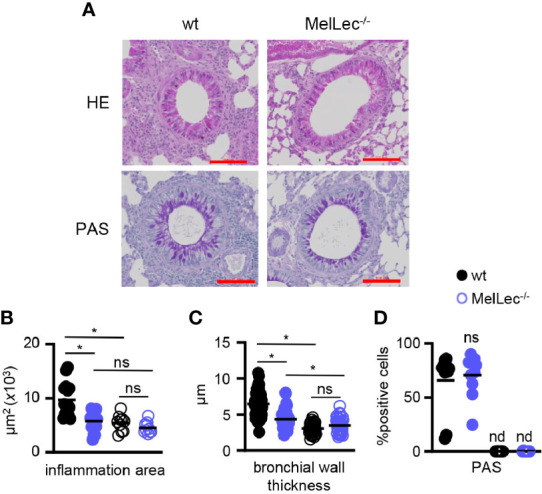
MelLec-deficient mice have reduced pulmonary pathology. **(A)** Representative Haematoxylin and Eosin (H&E) (top panels) and Periodic Acid Schiff (PAS) (bottom panels) stained lung sections from wild type (wt) and MelLec^-/-^ mice, as indicated, on day 23 following allergic sensitization and challenge with live *Aspergillus fumigatus* conidia. Scale bars represent 100 μm. Histological quantification of bronchial **(B)** inflammation (n=3; WT means 8644, 11892 and 7880 μm^2^; KO means 7104, 6176, 4081 μm^2^; mean difference is n.s.), **(C)** wall thickness (n=3; WT means 7.0, 7.4 and 6.1μm; KO means 5.2, 3.5, 4.0 μm; mean difference p<0.05) and **(D)** goblet cell hyperplasia (n=2; WT means 53.1 and 78.4%; KO means 74.5 and 66.9%), versus naïve mice (n=2). Shown are pooled data (with mean) of five representative bronchioles from the indicated number of mice. *p<0.05, n.s., not significant, determined from pooling the data of all samples from the mice. Statistical significance was determined using ANOVA **(B, C)** and student t-test **(D)**.

We next performed histological analysis of lungs of wt and MelLec^-/-^ mice on day 23, following the last challenge with *A. fumigatus* conidia (**Figure 1A**). As expected (5, 10), wt mice presented with inflammatory foci around bronchioles and increased bronchial wall thickening (**Figures 2A–C**). In contrast, MelLec-deficient mice presented with significantly less pulmonary inflammation and bronchial wall thickening (**Figures 2A–C**), indicative of a substantially reduced inflammatory response in these animals. On the other hand, using PAS staining, we found no differences between wt and MelLec^-/-^ mice in mucus-producing goblet cells (**Figures 2A, D**), which are induced as part of the allergic response (10).

### MelLec Regulates Neutrophil, Inflammatory Monocyte and T-Cell Recruitment During *A. fumigatus*-Induced Allergic Inflammation

**Figure 3:**
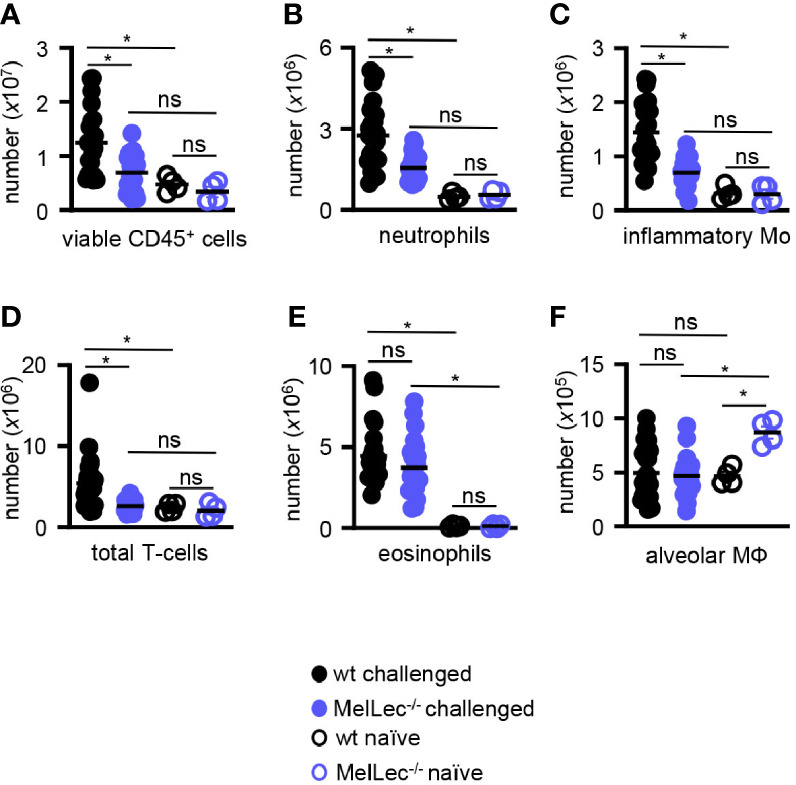
Loss of MelLec leads to reduced pulmonary cellular inflammation. **(A)** Total CD45^+^ leukocytes, **(B)** neutrophils (Ly6G^+^CD11b^+^), **(C)** inflammatory macrophage/monocytes (Ly6c^+^CD11b^+^), **(D)** total T-cells (CD11b^-^CD11c^-^MHCII^-^), **(E)** eosinophils (SIGLEC-F^+^CD11b^+^) and **(F)** alveolar macrophages (SIGLEC-F^+^CD11c^+^) in the lungs of wild type (wt) and MelLec^-/-^ mice, as indicated, on day 23 following allergic sensitization and challenge with live *Aspergillus fumigatus* conidia. Cellular counts from naïve mice are also shown (n=4). See **Supplementary Figure 1** for gating strategy). Shown are pooled data (with mean) from at least two independent experiments (n>20 mice). *p<0.05. n.s., not significant. Statistical significance was determined using ANOVA.

We then analyzed the composition of cellular infiltrate in the lungs of challenged mice by flow cytometry (**Supplementary Figure 1**). Consistent with our histological observations, described above, we observed significantly reduced numbers of total viable CD45^+^ myeloid cells in the lungs of the knock-out mice compared to wt animals (**Figure 3A**). This reduced cellular inflammation correlated with a reduced influx of CD11b^+^Ly6G^+^ neutrophils (**Figure 3B**), CD11b^+^Ly6C^high^ inflammatory monocytes (**Figure 3C**) and T-cells (**Figure 3D**). Notably, wt and MelLec^-/-^ mice presented with similar numbers of CD11b^+^Siglec-F^+^ eosinophils (**Figure 3E**), and also had similar numbers of CD11c^+^Siglec-F^+^ alveolar macrophages (**Figure 3F**). There were no differences in inflammatory cell numbers in the lungs of naïve wt and MelLec^-/-^ mice, although the knockout mice possessed higher basal levels of alveolar macrophages (**Figure 3F**).

### MelLec Influences Cytokine Responses During Allergic Inflammation

**Figure 4:**
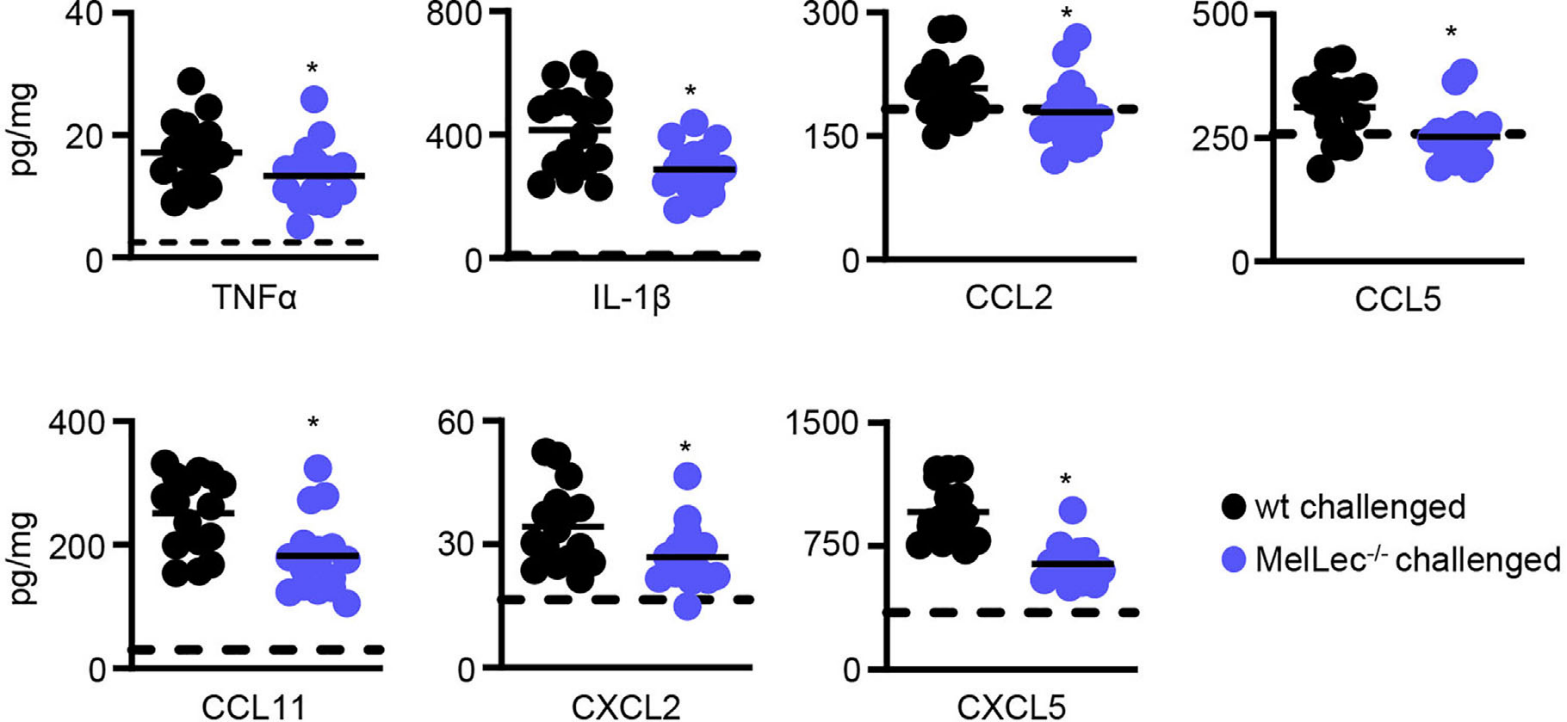
Loss of MelLec leads to reduced pulmonary cytokine and chemokine levels. Cytokine and chemokine levels in the lungs of wild type (wt) and MelLec^-/-^ mice on day 23 following sensitization and challenge with live *Aspergillus fumigatus* conidia. Shown are pooled data (with mean) from at least two independent experiments (n>15 mice). *p<0.05. Statistical significance between wt and MelLec^-/-^ animals were determined using t-tests. Data from naïve mice are shown in **Supplementary Figure 4**.

To gain insight into underlying reasons for the reduced cellular inflammation in the MelLec-deficient animals, we determined pulmonary inflammatory cytokine and chemokine responses. Compared to wt animals, MelLec-deficient mice presented with significantly lower levels of multiple inflammatory cytokines and chemokines including TNFα, IL-1β, CCL2, CCL5, CCL11, CXCL2 and CXCL5 (**Figure 4**). Data from naïve mice are shown in **Supplementary Figure 4**.

### Loss of MelLec Leads to Reduced Th17 Responses

**Figure 5:**
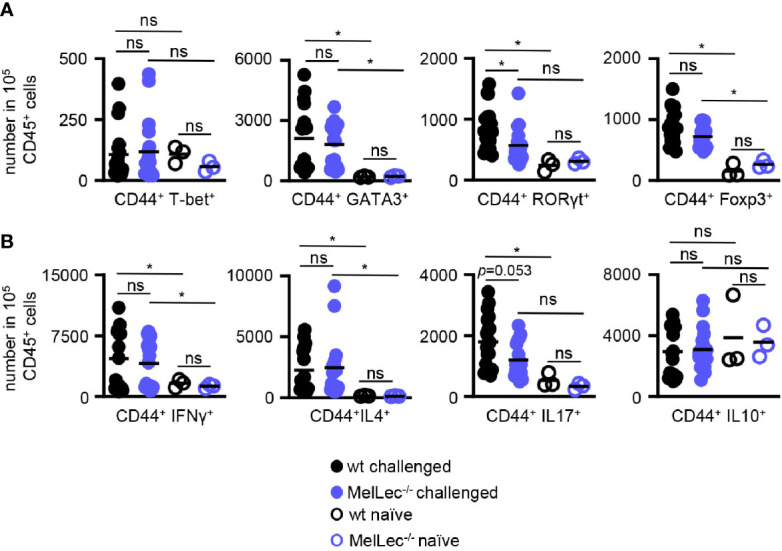
Altered T-cell responses in MelLec-deficient mice. Flow cytometric analysis of T-cell expressed **(A)** transcription factors and **(B)** cytokines, as indicated, in the lungs of wild type (wt) and MelLec^-/-^ mice on day 23 following sensitization and challenge with live *Aspergillus fumigatus* conidia. CD44^+^CD4^+^ T cells expressing transcription factors or cytokines are expressed as numbers per 1x10^5^ CD45^+^ cells. Shown are pooled data (with mean) from at least two independent experiments (n>15 mice). Data from unchallenged naïve mice are also shown (n=3). *p<0.05. n.s., not significant. Statistical significance was determined using ANOVA.

Finally, using flow cytometry (**Supplementary Figure 2**), we determined if loss of MelLec influenced the development of pulmonary CD4^+^ T-cell responses in our allergic model. In comparison to wt mice, we observed no alterations in the levels of expression of GATA3 and FoxP3 in activated T-cells from MelLec-deficient animals, indicating that similar Th2 and Treg responses were being induced similarly in both groups (**Figure 5A**). In contrast, there were significantly fewer RORγT^+^ T-cells in the knockout animals. There was no significant induction of T-bet in wt or MelLec-deficient mice, in this model (**Figure 5A**). Analysis of activated cytokine producing T-cells suggested that IL-17 producing cells were reduced (**Figure 5B**), although the difference was not statistically significant (*p*=0.053). Activated IL4^+^ and IFNγ^+^ producing T-cells were similarly induced in both wt and MelLec-deficient mice. We did not detect significant induction of IL-10^+^ -producing T-cells in either group of mice, compared to naïve animals (**Figure 5B**).

## Discussion

Asthma is a common lung disease characterized by chronic airway inflammation. Genetics and environmental factors are related to pathogenesis, with major environmental risk factors including inhaled substances and particles (such as fungi) that provoke allergic reactions (11). While the link between fungal sensitization and asthma pathogenesis is well established (1), the mechanisms by which fungi influence pulmonary allergic responses are still unclear. Several studies have linked the development of allergic responses to fungal cell wall components such as β-glucan or mannan, which induce immune responses through receptors including CLRs such as Dectin-1 (3). Many fungal pathogens linked to asthma, such as *Aspergillus* spp., *Alternaria* spp., or *Cladosporium* spp. also produce DHN-melanin, which has been suggested to modulate pulmonary inflammation in mouse models of asthma (12). In fungi, melanin has been implicated in diverse functions, including radiation protection, immune defense, and virulence (13).

We recently identified a novel CLR, MelLec, which recognizes fungal DHN-melanin and was essential for resistance to systemic infection with *A. fumigatus* (6). Loss of MelLec led to increased fungal burdens and alterations in inflammatory responses, from which the animals succumbed (6). In that study, we also showed that MelLec-deficient mice challenged with a single intratracheal dose of *A. fumigatus* conidia presented with reduced pulmonary inflammatory responses at 4hr following challenge (6), although the inflammation reverted to the levels found in the wt mice by 24hr (6). Notably, loss of MelLec did not alter the susceptibility of mice to pulmonary infection (6).

Given that *A. fumigatus* is thought to be one of the most important fungal organisms involved in the pathogenesis of asthma (1), we explored how MelLec influenced the development of pulmonary allergic responses with this pathogen in a mouse model. Unexpectedly, we found that MelLec-deficiency was protective. In contrast to wt animals, MelLec^-/-^ mice showed no adverse effects on weight gain. In fact, these animals gained weight at a rate equivalent to uninfected knockout or wt animals. There were also no alterations in the ability of MelLec-deficient mice to survive infection following repeated administration of conidia, similar to what we had observed following a single challenge (6). Thus, MelLec appears to be dispensable for resistance to pulmonary infection with *A. fumigatus* in immunocompetent animals, even after repeated challenges.

The protective effect of MelLec-deficiency is likely to be due to the significantly reduced pulmonary inflammatory responses in these animals. Indeed, loss of MelLec was associated with reduced cellular influx into the lungs, particularly neutrophils and inflammatory monocytes, and correlated with reduced levels of multiple inflammatory cytokine and chemokines. The impact on neutrophil recruitment is consistent with our previous observations following a single pulmonary challenge with conidia (6). Moreover, our data are also consistent with a previous report demonstrating reduced pulmonary neutrophil responses in wild type mice challenged with *A. fumigatus Δalb1*, which lack melanin, compared to wild type mice challenged with melanized conidia (12). This reduced cellular inflammation also correlated with our histological analysis. However, we note that the difference in pulmonary inflammation, as determined by histology, was only apparent when we pooled measurements of each individual bronchiole from the animals sampled. There was no significant difference in inflammation by histology, when the means of each animal were compared, indicating that the study was underpowered. Nevertheless, the cumulative evidence from the flow cytometry analyses of cellular influx, the cytokine analyses, and histological assessments indicate that MelLec^-/-^ mice have reduced pulmonary inflammation in this allergic model.

Surprisingly, knockout animals had ~0.5 log higher fungal lung burdens compared to wt animals, yet displayed no obvious adverse clinical effects. This indicates that although MelLec is required to control fungal burden in the lungs, as we have noted previously (6), the inflammatory responses mediated by the receptor in this allergic model negatively impact welfare of the animals. Interestingly, in a immunocompromised (cyclophosphamide) mouse model of pulmonary infection with a single dose (10^6^) of *A. fumigatus* conidia, there was also a reproducible, but non-significant, trend towards increased survival and higher fungal burdens in the MelLec^-/-^ animals (Stappers et al., personal communication). Such increased fungal burdens are likely to have a physiological impact that has yet to be revealed.

The alterations in neutrophil recruitment suggested that MelLec may influence the development of Th17 immunity. Th17 responses are associated with asthma pathogenesis, especially in corticosteroid resistant refractory asthma where neutrophils play a critical role (14). Moreover, the use of inhaled corticosteroids can lead to increased fungal burdens and deterioration of asthma control (15). We found a significant reduction in the number of activated Th17 cells (CD44^+^RORγt^+^) in the lungs of MelLec-deficient animals, suggesting that this CLR promotes the development of Th17 immunity. This correlated with a reduced number of CD44^+^IL17^+^ T-cells in MelLec-deficient animals, although this result was not statistically significant (*p*=0.053). Th2 responses were largely unaffected in the MelLec^-/-^ mice, evidenced by equivalent eosinophil recruitment, equivalent goblet cell hyperplasia, and equivalent Th2 T-cells in the lungs of these animals. The induction of Th1 and Treg responses appeared unaffected by MelLec deficiency. Our data is in contrast to a previous report that suggested that MelLec functions to suppress Th17 and Th1 responses, but this difference is likely due the different (non-pulmonary) experimental immunization protocol and the different rodent species used in that study (7).

How MelLec mediates its functions are still unclear. The cytoplasmic tail of MelLec lacks recognizable signaling motifs (6), and the receptor is primarily expressed by endothelial cells in mice. Although endothelial cells do contribute to asthma pathogenesis (16), it is still unclear how MelLec is able to sense fungal melanin in the endothelium following airway administration of conidia. One possibility could be the shedding of melanin, once the conidia begin to swell and germinate (17). Moreover, while other pattern recognition receptors, such as protease-activated receptor 2 (PAR-2), play an active role in mediating endothelial-mediated allergic responses (16), it is unclear how MelLec functions in this context. MelLec is likely to contribute to the pathogenesis of asthma in humans, as we have shown that the human receptor recognizes DHN-melanin and plays a role in anti-*Aspergillus* immunity (6). Notably, we discovered a common polymorphism of this receptor that increases susceptibility to systemic infections with *Aspergillus*, in part through reduced inflammatory responses (6). It is tempting to speculate that the alterations in MelLec function associated with this polymorphism (6), may protect against allergic pulmonary responses to melanized fungi in humans as we have seen in our mouse model. How MelLec mediates its activities and its contribution to the pathogenesis of human asthma is the focus of our future studies.

## Data Availability Statement

The original contributions presented in the study are included in the article/**Supplementary Material**. Further inquiries can be directed to the corresponding author.

## Ethics Statement

All animal use was approved and in compliance with local University animal research ethical regulations and a UK Home Office project licence (P79B6F297).

## Author Contributions

KT, MS, RH, ID, FS, and CW performed the experiments and analyzed the results. RY, PM, KK, JW, and GB provided essential input into experimental design and analysis of results. All authors contributed to the article and approved the submitted version.

## Conflict of Interest

The authors declare that the research was conducted in the absence of any commercial or financial relationships that could be construed as a potential conflict of interest.

## Abbreviations

MelLec, melanin-sensing C-type lectin receptor; DHN melanin, 1,8-dihydroxynaphthalene melanin; wt, wild-type.

## References

[1] Knutsen AP Bush RK Demain JG Denning DW Dixit A Fairs A. Fungi and Allergic Lower Respiratory Tract Diseases. J Allergy Clin Immunol (2012) 129:280–291; quiz 292-283. 10.1016/j.jaci.2011.12.970 22284927

[2] Denning DW O’driscoll BR Powell G Chew F Atherton GT Vyas A. Randomized Controlled Trial of Oral Antifungal Treatment for Severe Asthma With Fungal Sensitization: The Fungal Asthma Sensitization Trial (FAST) Study. Am J Respir Crit Care Med (2009) 179:11–8. 10.1164/rccm.200805-737OC 18948425

[3] Hadebe S Brombacher F Brown GD. C-Type Lectin Receptors in Asthma. Front Immunol (2018) 9:733. 10.3389/fimmu.2018.00733 29696023 PMC5904212

[4] Brown GD Willment JA Whitehead L. C-Type Lectins in Immunity and Homeostasis. Nat Rev Immunol (2018) 18:374–89. 10.1038/s41577-018-0004-8 29581532

[5] Hadebe S Kirstein F Fierens K Chen K Drummond RA Vautier S. Microbial Ligand Costimulation Drives Neutrophilic Steroid-Refractory Asthma. PLoS One (2015) 10:e0134219. 10.1371/journal.pone.0134219 26261989 PMC4532492

[6] Stappers MHT Clark AE Aimanianda V Bidula S Reid DM Asamaphan P. Recognition of DHN-melanin by a C-type Lectin Receptor is Required for Immunity to Aspergillus. Nature (2018) 555:382–6. 10.1038/nature25974 PMC5857201 29489751

[7] Lopez Robles MD Pallier A Huchet V Le Texier L Remy S Braudeau C. Cell-Surface C-type Lectin-Like Receptor CLEC-1 Dampens Dendritic Cell Activation and Downstream Th17 Responses. Blood Adv (2017) 1:557–68. 10.1182/bloodadvances.2016002360 PMC5728597 29296975

[8] Shafiei-Jahani P Helou DG Hurrell BP Galle-Treger L Howard E Quach C. CD52-Targeted Depletion by Alemtuzumab Ameliorates Allergic Airway Hyperreactivity and Lung Inflammation. Mucosal Immunol (2021). 10.1038/s41385-021-00388-5 PMC8225558 33731828

[9] Percopo CM Brenner TA Ma M Kraemer LS Hakeem RM Lee JJ. SiglecF+Gr1hi Eosinophils are a Distinct Subpopulation within the Lungs of Allergen-Challenged Mice. J Leukoc Biol (2017) 101:321–8. 10.1189/jlb.3A0416-166R PMC5166438 27531929

[10] Lilly LM Gessner MA Dunaway CW Metz AE Schwiebert L Weaver CT. The Beta-Glucan Receptor Dectin-1 Promotes Lung Immunopathology During Fungal Allergy Via IL-22. J Immunol (2012) 189:3653–60. 10.4049/jimmunol.1201797 PMC3448838 22933634

[11] Papi A Brightling C Pedersen SE Reddel HK. Asthma. Lancet (2018) 391:783–800. 10.1016/S0140-6736(17)33311-1 29273246

[12] Buskirk AD Templeton SP Nayak AP Hettick JM Law BF Green BJ. Pulmonary Immune Responses to Aspergillus Fumigatus in an Immunocompetent Mouse Model of Repeated Exposures. J Immunotoxicol (2014) 11:180–9. 10.3109/1547691X.2013.819054 PMC4604601 23919459

[13] Smith DFQ Casadevall A. The Role of Melanin in Fungal Pathogenesis for Animal Hosts. Curr Top Microbiol Immunol (2019) 422:1–30. 10.1007/82_2019_173 31278515

[14] Mckinley L Alcorn JF Peterson A Dupont RB Kapadia S Logar A. TH17 Cells Mediate Steroid-Resistant Airway Inflammation and Airway Hyperresponsiveness in Mice. J Immunol (2008) 181:4089–97. 10.4049/jimmunol.181.6.4089 PMC3638757 18768865

[15] Fraczek MG Chishimba L Niven RM Bromley M Simpson A Smyth L. Corticosteroid Treatment is Associated with Increased Filamentous Fungal Burden in Allergic Fungal Disease. J Allergy Clin Immunol (2018) 142:407–14. 10.1016/j.jaci.2017.09.039 29122659

[16] Asosingh K Weiss K Queisser K Wanner N Yin M Aronica M. Endothelial Cells in the Innate Response to Allergens and Initiation of Atopic Asthma. J Clin Invest (2018) 128:3116–28. 10.1172/JCI97720 PMC6025973 29911993

[17] Latge JP Beauvais A Chamilos G. The Cell Wall of the Human Fungal Pathogen Aspergillus Fumigatus: Biosynthesis, Organization, Immune Response, and Virulence. Annu Rev Microbiol (2017) 71:99–116. 10.1146/annurev-micro-030117-020406 28701066

