# Supplementary material for: MelLec Exacerbates the Pathogenesis of Aspergillus fumigatus-Induced Allergic Inflammation in Mice
Source: Front Immunol. 2021 May 28;12:675702. doi: 10.3389/fimmu.2021.675702 (PMC8194280; doi:10.3389/fimmu.2021.675702)
Supplement: Supplementary file 1 [file DataSheet_1.pdf]

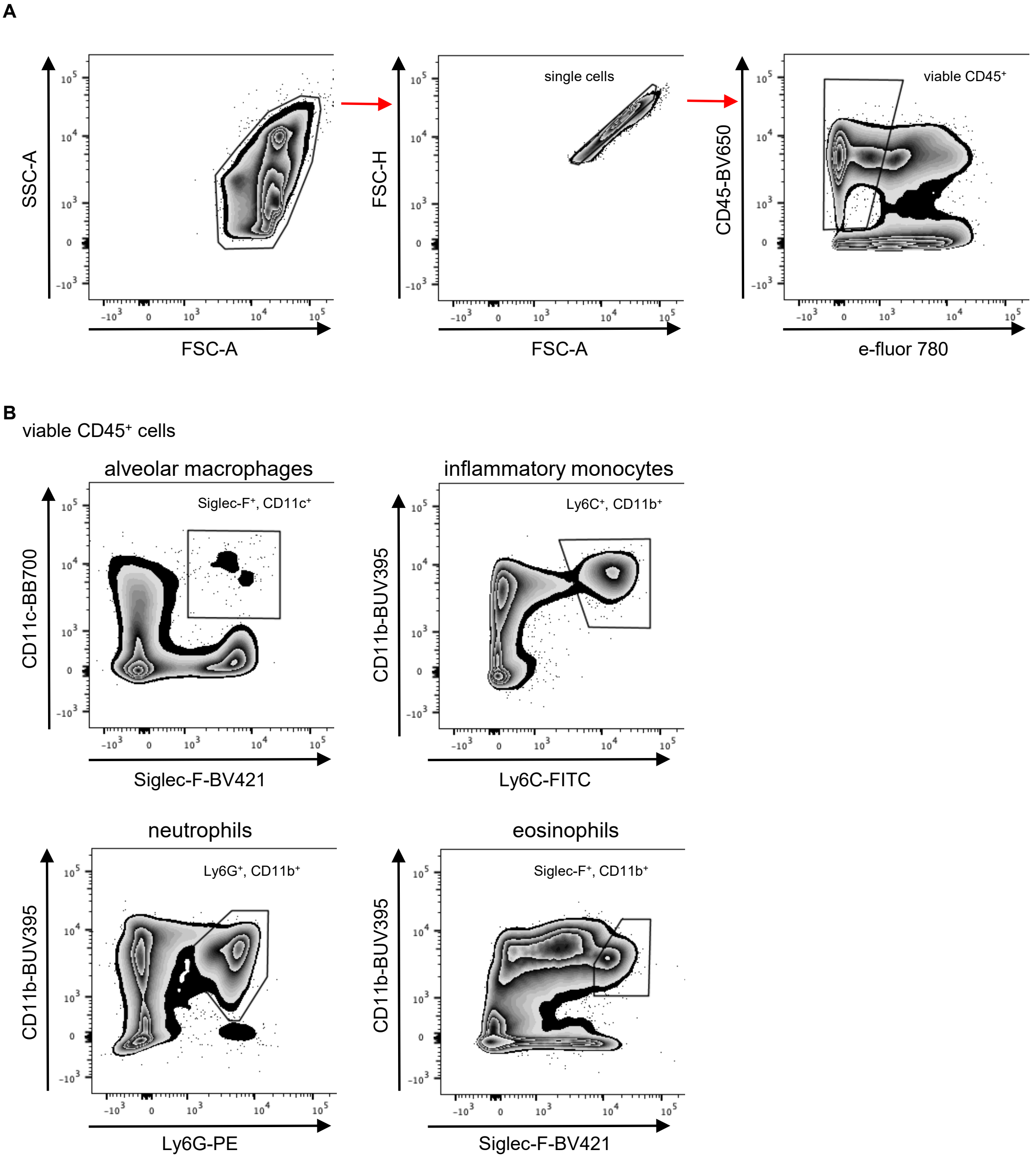

Supporting information Figure 1: Flow cytometry gating strategy for the identification of cells from the murine lung.

(**A**) Gating strategy for the identification of single, live, CD45$^+$ cells. (**B**) Gating strategy on viable CD45$^+$ cells used to identify alveolar macrophage, inflammatory monocytes, neutrophils and eosinophils, as indicated. This broad gating strategy to identify cell types was selected based on previously published approaches (Hadebe et al., 2015;Shafiei-Jahani et al., 2021). However, some of the markers combinations used (eg: Ly6C, Ly6G, CD11b and CD11c) are not definitive and our analyses do not fully exclude small elements of contamination between cellular populations (Percopo et al., 2017).

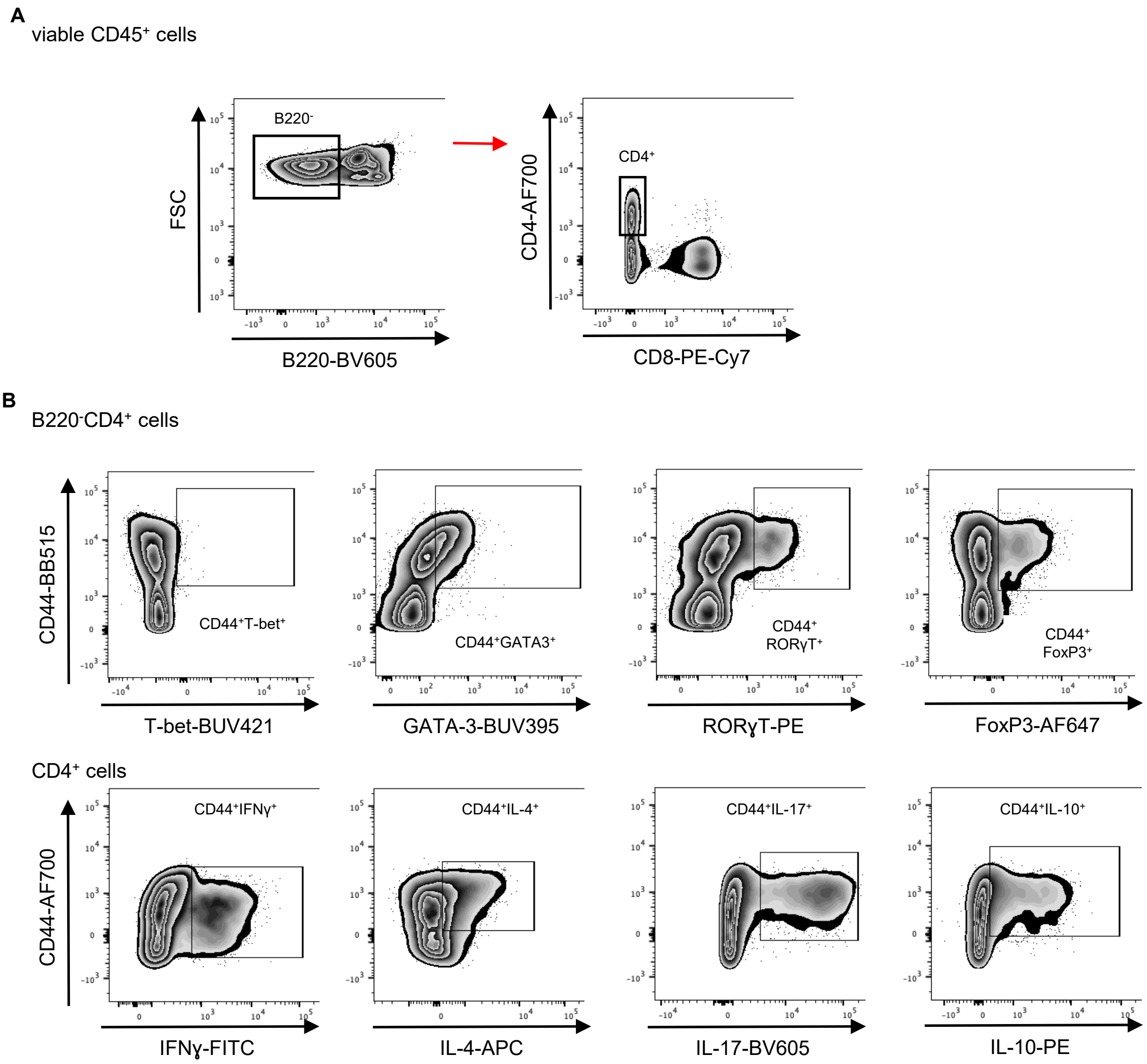

Supporting information Figure 2: Flow cytometry gating strategy for the characterization of T-cells the murine lung.

(**A**) Gating strategy to identify B220$^-$CD8$^-$CD4$^+$ T-cells from single, live, CD45$^+$ cells (Supplemental Figure 1). (**B**) Gating strategy on B220$^-$CD8$^-$CD4$^+$ T-cells to characterize transcription factors and cytokines, as indicated.

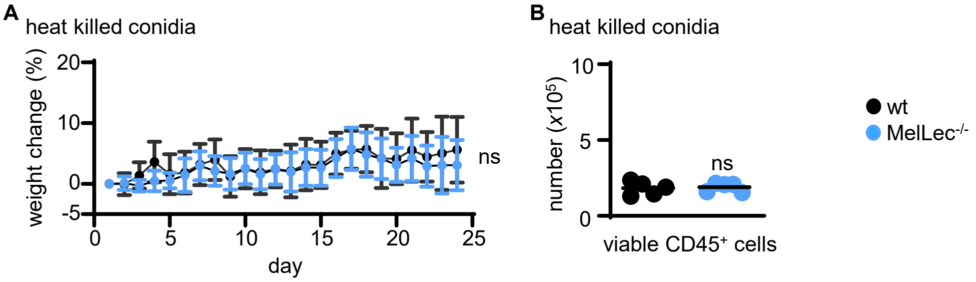

Supporting information Figure 3: Impact of MelLec requires live conidia.
Percentage body weight change (**A**) and total bronchoalveolar lavage $CD45^+$ cells (B) in naïve wild type (wt) or $MelLec^{-/-}$ mice following sensitisation and challenge with heat-killed Aspergillus fumigatus conidia. Values are mean ± SD of one experiment (n=5 mice). Statistical significance was determined using t-tests or Mann–Whitney U test.

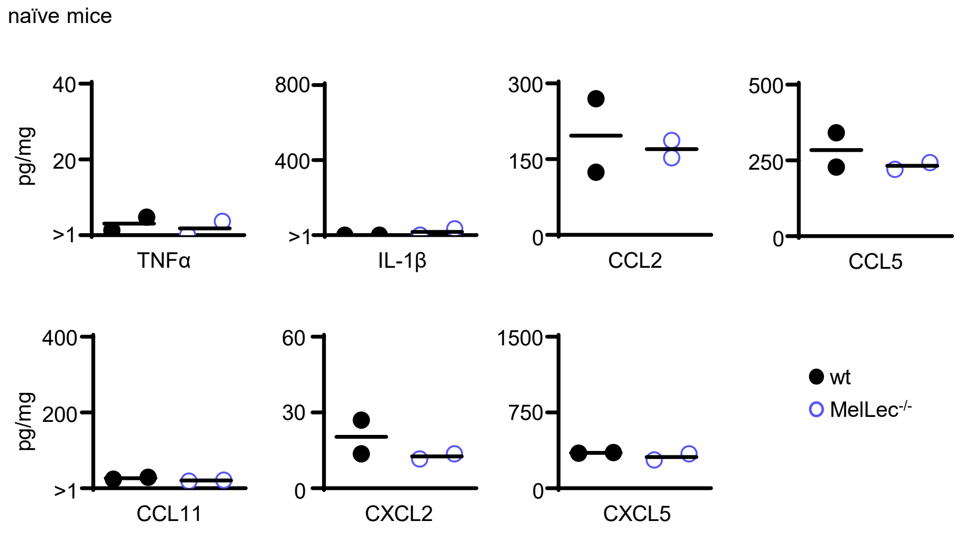

Supporting information Figure 4: Pulmonary cytokine and chemokine levels in naïve mice. Cytokine and chemokine levels in the lungs of naïve wild type (wt) and MelLec-/- mice (n=2). >1; below limits of detection.
